# Supplementary material for: PEGDA microencapsulated allogeneic islets reverse canine diabetes without immunosuppression
Source: PLoS One. 2022 May 25;17(5):e0267814. doi: 10.1371/journal.pone.0267814 (PMC9132281; doi:10.1371/journal.pone.0267814)
Supplement: S1 File — (DOCX) [file pone.0267814.s001.docx]

**Supplemental Table 2: Conversion of Canine Islet Diameters to Cell Numbers.**

Canine islets from 3 donors were isolated and a total of 450 islets were manually placed in single wells of 384 well plates with 20 μL of CMRL media/well (one islet/well). In order to create the correlation between diameter and cell number, islets were chosen to cover a wide range of sizes, thus they do not represent the natural distribution of canine islet sizes.

The diameter in the X and Y directions and the total perimeter of each individual islet was measured on a Zeiss AXIO inverted microscope with a Jenoptick C3 camera using Capture Pro V2.8.8 software. Each islet was dispersed into single cells by exposure to trypsin and manual pipetting. Individual cells per well were counted on a Biotek Cytation 5 Imaging microplate reader. The average diameter (X and Y) are shown in the highlighted columns with the corresponding cell number in the following column.

| Diameter | Cell Number | Diameter | Cell Number | Diameter | Cell Number |
| --- | --- | --- | --- | --- | --- |
| 128.0 | 98 | 95 | 57 | 153.5 | 375 |
| 119 | 212 | 126 | 221 | 106 | 127 |
| 64 | 45 | 155.5 | 296 | 90 | 99 |
| 135 | 242 | 116.5 | 156 | 86.5 | 113 |
| 85.5 | 18 | 94 | 97 | 96.5 | 223 |
| 172.5 | 769 | 126.5 | 212 | 133 | 238 |
| 119 | 124 | 105.5 | 137 | 131 | 175 |
| 83 | 129 | 148 | 439 | 120.5 | 136 |
| 116.5 | 323 | 137 | 357 | 116 | 172 |
| 155 | 401 | 126 | 209 | 107.5 | 203 |
| 85.5 | 129 | 104 | 54 | 136 | 321 |
| 103 | 237 | 78 | 176 | 118.5 | 191 |
| 160 | 223 | 129 | 27 | 83.5 | 131 |
| 70 | 36 | 51 | 48 | 104 | 152 |
| 103 | 110 | 76 | 267 | 106 | 140 |
| 180 | 340 | 154 | 378 | 133.5 | 219 |
| 62 | 17 | 134 | 299 | 123 | 233 |
| 88 | 76 | 147 | 256 | 104 | 191 |
| 92 | 103 | 134 | 427 | 134 | 320 |
| 134 | 331 | 117.5 | 175 | 103.5 | 206 |
| 157 | 369 | 80 | 62 | 122.5 | 270 |
| 147 | 558 | 78 | 79 | 70.5 | 70 |
| 143 | 310 | 72 | 61 | 148 | 374 |
| 67 | 50 | 167 | 658 | 47 | 11 |
| 103 | 58 | 121 | 248 | 90 | 9 |
| 140 | 336 | 54 | 10 | 70.5 | 77 |
| 114 | 24 | 109 | 324 | 66.5 | 74 |
| 102 | 95 | 82 | 160 | 64 | 56 |
| 74.5 | 99 | 98 | 336 | 72.5 | 74 |
| 115.5 | 154 | 59.5 | 48 | 74.5 | 107 |
| 90.5 | 41 | 109 | 151 | 133.5 | 368 |
| 77 | 95 | 206 | 809 | 181.5 | 984 |
| 102 | 10 | 104 | 224 | 102 | 208 |
| 83 | 147 | 146 | 655 | 81.5 | 119 |
| 63 | 46 | 115.5 | 389 | 118.5 | 381 |
| 141 | 395 | 91.5 | 264 | 170 | 568 |
| 77 | 142 | 98.5 | 191 | 136 | 445 |
| 101 | 184 | 202.5 | 842 | 87.5 | 144 |
| 93 | 236 | 70 | 81 | 71 | 120 |
| 144.5 | 681 | 120 | 393 | 120 | 195 |
| 124 | 453 | 150.5 | 727 | 78 | 104 |
| 87 | 126 | 112 | 322 | 174 | 653 |
| 77 | 99 | 144.5 | 410 | 130 | 424 |
| 59 | 12 | 67 | 80 | 76 | 116 |
| 99.5 | 189 | 92 | 199 | 141 | 392 |
| 163 | 544 | 101.5 | 384 | 67 | 175 |
| 52 | 84 | 112 | 36 | 82 | 19 |
| 132 | 100 | 71.5 | 212 | 163.5 | 457 |
| 104.5 | 288 | 173.5 | 1017 | 135 | 606 |
| 96 | 165 | 115 | 339 | 103 | 418 |
| 43 | 115 | 121.5 | 450 | 112 | 269 |
| 162 | 619 | 205.5 | 764 | 156.5 | 613 |
| 219.5 | 799 | 130 | 309 | 105.5 | 333 |
| 186 | 211 | 198.5 | 522 | 182 | 1006 |
| 81.5 | 139 | 164.5 | 450 | 75 | 267 |
| 109 | 93 | 90 | 65 | 126 | 489 |
| 119.5 | 139 | 112 | 56 | 97.5 | 269 |
| 70 | 83 | 116.5 | 408 | 143.5 | 833 |
| 132.5 | 195 | 73.5 | 51 | 188.5 | 1484 |
| 170.5 | 436 | 121 | 331 | 114 | 372 |
| 109 | 196 | 107 | 66 | 149.5 | 630 |
| 173 | 454 | 122 | 470 | 145.5 | 781 |
| 113 | 234 | 142.5 | 104 | 53.5 | 28 |
| 95 | 355 | 168 | 612 | 169 | 323 |
| 139.5 | 406 | 117.5 | 225 | 114.5 | 193 |
| 141 | 688 | 232 | 1237 | 148.5 | 368 |
| 137 | 752 | 155 | 261 | 103 | 249 |
| 112 | 196 | 175 | 876 | 71.5 | 302 |
| 132.5 | 670 | 147.5 | 363 | 140.5 | 263 |
| 222 | 347 | 138.5 | 223 | 136 | 906 |
| 225 | 314 | 58 | 47 | 161.5 | 540 |
| 166 | 342 | 132.5 | 394 | 105 | 367 |
| 166.5 | 434 | 129.5 | 511 | 164.5 | 921 |
| 127 | 201 | 219.5 | 499 | 112.5 | 263 |
| 177.5 | 589 | 189.5 | 534 | 83 | 169 |
| 181 | 50 | 121.5 | 646 | 185.5 | 1209 |
| 208 | 531 | 104.5 | 443 | 196.5 | 951 |
| 152.5 | 498 | 142.5 | 382 | 223 | 389 |
| 183.5 | 581 | 181 | 495 | 211 | 1103 |
| 188.5 | 681 | 169 | 292 | 150.5 | 295 |
| 188.5 | 389 | 244.5 | 612 | 127.5 | 371 |
| 181 | 601 | 133.5 | 605 | 194 | 725 |
| 218.5 | 884 | 192.5 | 990 | 157.5 | 723 |
| 143 | 205 | 248 | 901 | 190.5 | 846 |
| 159 | 591 | 154 | 915 | 145 | 508 |
| 132.5 | 739 | 208.5 | 827 | 152 | 981 |
| 170.5 | 990 | 260.5 | 1454 | 132 | 346 |
| 135.5 | 372 | 226 | 915 | 216.5 | 394 |
| 224 | 1445 | 226 | 780 | 159.5 | 340 |
| 138 | 286 | 218 | 666 | 150.5 | 322 |
| 164 | 556 | 286 | 944 | 160.5 | 203 |
| 168.5 | 376 | 169 | 718 | 112 | 329 |
| 190.5 | 533 | 276 | 807 | 180 | 596 |
| 196 | 562 | 222.5 | 618 | 187.5 | 926 |
| 149.5 | 411 | 211.5 | 729 | 176.5 | 340 |
| 159.5 | 266 | 207.5 | 654 | 234.5 | 676 |
| 159.5 | 317 | 194.5 | 823 | 145.5 | 185 |
| 187.5 | 257 | 168.5 | 516 | 189.5 | 553 |
| 174 | 498 | 144.5 | 130 | 217.5 | 901 |
| 187.5 | 551 | 246.5 | 894 | 145 | 278 |
| 169 | 456 | 227.5 | 574 | 178.5 | 592 |
| 76.5 | 50 | 219.5 | 389 | 195 | 689 |
| 153 | 361 | 148.5 | 541 | 181.5 | 767 |
| 102 | 81 | 159 | 152 | 138.5 | 319 |
| 66.5 | 130 | 152 | 110 | 157.5 | 276 |
| 136.5 | 399 | 135 | 241 | 217.5 | 591 |
| 88.5 | 152 | 121 | 86 | 140 | 219 |
| 126.5 | 193 | 122.5 | 262 | 114.5 | 355 |
| 53 | 51 | 133.5 | 141 | 80.5 | 68 |
| 154 | 99 | 56.5 | 54 | 95.5 | 87 |
| 119.5 | 109 | 116.5 | 95 | 59.5 | 86 |
| 107 | 102 | 211.5 | 472 | 54 | 213 |
| 179.5 | 270 | 58 | 65 | 79 | 91 |
| 78.5 | 96 | 151 | 337 | 137 | 394 |
| 128 | 205 | 165 | 551 | 133 | 262 |
| 87 | 83 | 183 | 506 | 134.5 | 288 |
| 108.5 | 128 | 89 | 186 | 146.5 | 351 |
| 181.5 | 788 | 99.5 | 177 | 111.5 | 370 |
| 105 | 196 | 202 | 370 | 133.5 | 587 |
| 116.5 | 302 | 127 | 190 | 128 | 317 |
| 90 | 51 | 157.5 | 723 | 88 | 76 |
| 83 | 147 | 190.5 | 846 | 132.5 | 455 |
| 102 | 10 | 145 | 508 | 71 | 61 |
| 93 | 135 | 152 | 981 | 147 | 311 |
| 63 | 45 | 180 | 345 | 129.5 | 238 |
| 141 | 402 | 216.5 | 394 | 108 | 213 |
| 50.5 | 29 | 159.5 | 340 | 166.5 | 344 |
| 100 | 160 | 134 | 230 | 88.5 | 85 |
| 67.5 | 88 | 146 | 339 | 126 | 239 |
| 144.5 | 484 | 132 | 346 | 75 | 84 |
| 148 | 655 | 147 | 395 | 150.5 | 375 |
| 148.5 | 758 | 143 | 374 | 105 | 152 |
| 145.45 | 701 | 117 | 266 | 111 | 191 |
| 164 | 880 | 80 | 134 | 103 | 208 |
| 129 | 528 | 153 | 496 | 145.5 | 326 |
| 59 | 52 | 90 | 45 | 119.5 | 397 |
| 210 | 541 | 96 | 167 | 120 | 355 |
| 199 | 600 | 86 | 84 | 143 | 503 |
| 205.5 | 578 | 132 | 322 | 92 | 336 |
| 53.5 | 30 | 104 | 206 | 119 | 345 |
| 67.5 | 176 | 133 | 248 | 121 | 388 |
| 163 | 544 | 103 | 100 | 172.5 | 769 |
| 92 | 198 | 92.5 | 204 | 154.5 | 508 |
| 132 | 100 | 91.5 | 165 | 157 | 591 |
| 105 | 246 | 62.5 | 41 | 88 | 176 |
| 104 | 226 | 133 | 401 | 69 | 38 |
| 109 | 152 | 69 | 75 | 80 | 65 |
| 206 | 763 | 62 | 77 | 106.5 | 228 |
| 105 | 225 | 62 | 54 | 90 | 199 |

**Supplemental Table 2: Canine Islet Size.**

Canine islet diameters from each isolation were binned into their respective diameters by a trained technician. The values are the average of 3 samples. Isolations are identified according to the recipient dog. When repeated doses were administered the identification is represented by the dog ID number followed by 1 or 2 as the first or second transplant. The results demonstrate that the majority of canine islets were less than 50 μm in diameter.

| Islet Diameters (mm) | Cell Counts Binned by Diameter | | | | | | | | |
| --- | --- | --- | --- | --- | --- | --- | --- | --- | --- |
|  | 1 | 2 | 3 | 4 | 5-1 | 5-2 | 6 | 7-1 | 7-2 |
| <50 | 187.6 | 218.8 | 271 | 140.4 | 291.6 | 176.5 | 376 | 284.8 | 176.5 |
| 50-100 | 54.6 | 51.8 | 67.6 | 32 | 48.4 | 52.8 | 98.6 | 53 | 52.8 |
| 101-150 | 8.6 | 10 | 13.8 | 8.8 | 5.2 | 19.4 | 18.2 | 6.2 | 19.4 |
| 151-200 | 1.2 | 2.2 | 3.4 | .06 | 0.2 | 0.4 | 2.4 | 0.4 | 0.4 |
| 201-250 | 0 | 0 | 0.6 | 0 | 0 | 0 | 0.4 | 0 | 0 |
| 251-300 | 0 | 0 | 0 | 0 | 0 | 0 | 0 | 0 | 0 |
| 301-350 | 0 | 0 | 0 | 0 | 0 | 0 | 0.2 | 0 | 0 |
| >350 | 0 | 0 | 0 | 0 | 0 | 0 | 0 | 0 | 0 |

**Supplemental Table 3: Purity of Canine Islets.**

The purity of the preparations was determined with dithizone staining for isolations used in the safety arm of the study with healthy dogs. FluoZin 3 staining was used to identify islets in the preparations used for the efficacy arm of the study in diabetic dogs. Images were collected on a Biotek Cytation 5 Imaging Multi-Mode microplate reader and the number of dithizone or FluoZin-positive islets counted along with all cells in the brightfield mode. The percentage of dithizone or FluoZin-positive cells were calculated. A minimum of 4 samples from each isolation were measured. When repeated doses were administered the identification is represented by the dog ID number followed by 1 or 2 as the first or second transplant.

| Purity (% Dithizone-Positive Cells) | | | | Purity (% FluoZin 3-Positive Cells) | | | | |
| --- | --- | --- | --- | --- | --- | --- | --- | --- |
| 1 | 2 | 3 | 4 | 5-1 | 5-2 | 6 | 7-1 | 7-2 |
| 50 | 55 | 67 | 55 | 36.4 | 66.2 | 45.3 | 47.9 | 66.2 |
| 46 | 60 | 50 | 50 | 42.4 | 70.6 | 51.5 | 53.7 | 70.6 |
| 51 | 65 | 40 | 40 | 36.3 | 50.5 | 54.2 | 57.1 | 50.5 |
| 44 | 57 | 47 | 71 | 47.1 | 73.7 | 46.4 | 43.6 | 73.7 |
| 32 | 62 | 43 | 42 | - |  | - | 41.1 | - |

**Supplemental Table 4: Canine Islet Viability Prior to Encapsulation**.

Unencapsulated cells were incubated in calcein AM and propidium iodide for 30 minutes. Fluorescence was captured with a multi-mode plate reader. The area of calcein-stained (live) cells was divided by the total cell area (obtained with brightfield images of the same fields) resulting in the percentage of live cells. When repeated doses were administered the identification is represented by the dog ID number followed by 1 or 2 as the first or second transplant.

| Unencapsulated Islet Viability (% Live) | | | | | | | | |
| --- | --- | --- | --- | --- | --- | --- | --- | --- |
| 1 | 2 | 3 | 4 | 5 | 5-2 | 6 | 7 | 7-2 |
| 74 | 79 | 71 | 62 | 62 | 96 | 83 | 80 | 96 |
| 68 | 49 | 67 | 73 | 88 | 90 | 77 | 85 | 90 |
| 75 | 76 | 81 | 81 | 66 | 64 | 89 | 67 | 64 |
| 71 | 76 | 74 | 70 | 57 | 59 | 99 | 89 | 59 |
| 88 | 68 | 75 | 64 | 76 | 94 | 100 | 83 | 94 |
| 80 | 86 | 67 | 79 | 80 | 67 | 88 | 86 | 67 |
| 77 | 74 | 60 | 63 | 66 | 75 | 86 | 58 | 75 |
| - | 68 | 77 | 75 | 70 | 64 | - | 65 | 64 |

**Supplemental Table 5: Encapsulated Islet Viability Before and After Transplantation.**

After encapsulation, the same viability stains were utilized to determine viability before and after transplant. After transplantation, attempts were made to collect free microspheres. Dog #7 was the only time that sufficient microspheres could be retrieved without significant damage.

| Encapsulated Islet Viability (% Live) | | | |
| --- | --- | --- | --- |
| Pre-Transplant | | | Pos-Transplant |
| Dog 5 | Dog 6 | Dog 7 | Dog 7 |
| 62 | 88 | 69 | 75 |
| 94 | 78 | 76 | 82 |
| 87 | 91 | 84 | 49 |
| 75 | 76 | 71 | 58 |
| 72 | 97 | 69 | 82 |
| 82 | 93 | 71 | 56 |
| 59 | 98 | 74 | 99 |
| 88 | 71 | 69 | 67 |
| 99 | 85 | 61 | 74 |
| 85 | 78 | 90 | 71 |
| 74 | 86 | 83 | 64 |
| 64 | 75 | 62 | 65 |
| 59 | 55 | 61 | 78 |
| 58 | 79 | 71 | 43 |
| 42 | 84 | - | 44 |
| 80 | 77 | - | 61 |

**Supplemental Table 6: Glucose-Stimulated Insulin Secretion from Unencapsulated Canine Islets.**

Unencapsulated canine islets were exposed to glucose solutions in EBSS. Islets were first equilibrated to the low glucose condition of 2.8 mM for 1 h. Transwell inserts in a 24-well plate held approximately 22,000 cells and were used to transfer the islets to increasing concentrations of glucose. Supernatant media was collected after 1 hour in each concentration and stored at −80 **°**C until quantification was performed using Mercodia’s canine insulin ELISA kit. Missing data fell outside of the linear range of the ELISA calibration.

| Glucose Concentrations (mM) | | | | | | | | |
| --- | --- | --- | --- | --- | --- | --- | --- | --- |
|  | 2.8 | 5.6 | 11.2 | 16.8 | 22.4 | 28 | 33.6 | 50 |
| Rep 1 | - | 65 | 105 | 106 | 122 | 146 | 163 | 166 |
| Rep 2 | - | 82 | 125 | 110 | 141 | 179 | 215 | 164 |
| Rep 3 | 50 | 110 | 105 | 133 | 156 | - | 195 | - |
| Rep 4 | 88 | 84 | 85 | 112 | 106 | 111 | 146 | 155 |
| Rep 5 | 92 | 94 | 94 | 101 | 102 | 251 | 266 | - |

**Supplemental Table 7: ATP and Insulin Levels**

The GeneJET Genomic DNA Purification Kit was used following the manufacturer’s recommendations. Eluted DNA was then quantitated using the Quant-iT PicoGreen dsDNA Assay Kit according to manufacturer’s instructions. ATP levels were measured using a luminescent ATP CellTiter-Glo assay following the manufacturer’s instructions. Islets were distributed in 96 well plates in CMRL media. They were exposed to the CellTiter-Glo reagent and 15 min later luminescence read on a Cytation 5 Imaging Multi-Mode Reader. For dogs #5 and 7 repeated doses were administered, thus the batch is identified by the dog ID number followed by 1 or 2 for the first or second transplant.

| Transplant Number | 5-1 | 5-2 | 6 | 7-1 | 7-2 |
| --- | --- | --- | --- | --- | --- |
| ATP (nM) | 613 | 4611 | 1312 | 1379 | 4611 |
| Insulin (μg) | 446.4 | 2414.0 | 1178.7 | 460.9 | 2414.0 |
| Cells in Sample | 17,126,647 | 236,817,260 | 74,086,314 | 24,849,736 | 236,817,260 |

**Supplemental Table 8: Microsphere Diameters.**

Samples of microspheres containing canine islets were measured for each transplant. The average diameter was determined using the a multi-mode microplate reader and measured using Image J.

| Recipient Dog ID | Diameter (μm) | Batch Average Diameter (μm) |
| --- | --- | --- |
| #1 | 1300 | 1054 ± 56 |
|  | 850 |  |
|  | 790 |  |
|  | 1200 |  |
|  | 1000 |  |
|  | 1000 |  |
|  | 1100 |  |
|  | 1300 |  |
|  | 1100 |  |
|  | 900 |  |
| #2 | 600 | 1033 ± 97 |
|  | 800 |  |
|  | 1100 |  |
|  | 1300 |  |
|  | 900 |  |
|  | 700 |  |
|  | 1400 |  |
|  | 1300 |  |
|  | 1200 |  |
| #3 | 1100 | 930 ± 83 |
|  | 900 |  |
|  | 500 |  |
|  | 600 |  |
|  | 700 |  |
|  | 1100 |  |
|  | 1200 |  |
|  | 1300 |  |
|  | 900 |  |
| #4 | 1200 | 930 ± 70 |
|  | 900 |  |
|  | 1300 |  |
|  | 900 |  |
|  | 1100 |  |
|  | 1200 |  |
|  | 800 |  |
|  | 800 |  |
|  | 1100 |  |
|  | 600 |  |
|  | 700 |  |
|  | 600 |  |
| #5  Transplant 1 | 1100 | 1092 ± 33 |
|  | 1200 |  |
|  | 1200 |  |
|  | 1200 |  |
|  | 1000 |  |
|  | 950 |  |
|  | 1100 |  |
|  | 1000 |  |
|  | 1200 |  |
|  | 1300 |  |
|  | 1200 |  |
|  | 950 |  |
|  | 950 |  |
|  | 950 |  |
| #5  Transplant 2 | 1100 | 960 ± 36 |
|  | 800 |  |
|  | 950 |  |
|  | 1000 |  |
|  | 850 |  |
|  | 1050 |  |
|  | 1050 |  |
|  | 750 |  |
|  | 1100 |  |
|  | 1000 |  |
|  | 900 |  |
| #6 | 1000 | 911 ± 30 |
|  | 900 |  |
|  | 900 |  |
|  | 900 |  |
|  | 950 |  |
|  | 1000 |  |
|  | 1000 |  |
|  | 1000 |  |
|  | 1100 |  |
|  | 1000 |  |
|  | 1000 |  |
| #7  Transplant 1 | 1100 | 991 ± 31 |
|  | 900 |  |
|  | 800 |  |
|  | 900 |  |
|  | 1000 |  |
|  | 900 |  |
|  | 1100 |  |
|  | 1000 |  |
|  | 900 |  |
|  | 1100 |  |
|  | 1100 |  |
|  | 1100 |  |
| #7  Transplant 2 | 1000 | 960 ± 28 |
|  | 900 |  |
|  | 650 |  |
|  | 900 |  |
|  | 1000 |  |
|  | 1100 |  |
|  | 900 |  |
|  | 800 |  |
|  | 1000 |  |
|  | 1100 |  |
|  | 1000 |  |
|  | 1000 |  |
|  | 900 |  |
|  | 900 |  |
|  | 1100 |  |
|  | 1150 |  |
|  | 900 |  |
|  | 1000 |  |

**Supplemental Table 9: Clusters per Microsphere**

The number of individual cell clusters (islets) per microsphere were manually counted using a multi-mode plate reader with microscopic features and a 4X objective. When repeated doses were administered the identification is represented by the dog ID number followed by 1 or 2 as the first or second transplant.

| Islet Clusters/Microcapsule (cluster number) for Each Batch | | | | | | | | |
| --- | --- | --- | --- | --- | --- | --- | --- | --- |
| 1 | 2 | 3 | 4 | 5-1 | 5-2 | 6 | 7-1 | 7-2 |
| 10 | 8 | 8 | 26 | 22 | 25 | 13 | 16 | 22 |
| 12 | 3 | 15 | 7 | 18 | 17 | 19 | 14 | 16 |
| 14 | 15 | 11 | 15 | 26 | 15 | 14 | 23 | 16 |
| 13 | 17 | 10 | 8 | 10 | 13 | 17 | 32 | 12 |
| 9 | 16 | 16 | 5 | 5 | 12 | 12 | 21 | 16 |
| 12 | 14 | 7 | 16 | 19 | 13 | 12 | 14 | 11 |
| 14 | 15 | 8 | 11 | 19 | 25 | 12 | 15 | 10 |
| 8 | 4 | 11 | 9 | 14 | 15 | 18 | 22 | 14 |
| 14 | 12 | 7 | 12 | 11 | 19 | 18 | 10 | 15 |
| 13 | 14 | 10 | 25 | 27 | 19 | 10 | 24 | 21 |
| 15 | 15 | 7 | 18 | 22 | 17 | 18 | 19 | 12 |
| 15 | 21 | 8 | 8 | 19 | 20 | 16 | 12 | 13 |
| 19 | 5 | 7 | 7 | 11 | 14 | 10 | 19 | 20 |
| 8 | 16 | 28 | 16 | 16 | 28 | 11 | 18 | 19 |
| 10 | 13 | 25 | 13 | 4 | 16 | 20 | 8 | 18 |
| 13 | 19 | 15 | 13 | 12 | 21 | 22 | 14 | 14 |
| 9 | 20 | 4 | 11 | 17 | 20 | 12 | 16 | 15 |
| 4 | 13 | 6 | 18 | 13 | 17 | 17 | 10 | 17 |
| 21 | 7 | 19 | 13 | - | 15 | 12 | 12 | 12 |
| 9 | 9 | 13 | 12 | - | 26 | 17 | 18 | 9 |
| 8 | 10 | 14 | 14 | - | 18 | 8 | 10 | - |
| 13 | 14 | 6 | 10 | - | 17 | - | 15 | - |
| 12 | 14 | 24 | 16 | - | 22 | - | - | - |
| 17 | 13 | 17 | - | - | 25 | - | - | - |
| 11 | 12 | 8 | - | - | - | - | - | - |
| - | 3 | 16 | - | - | - | - | - | - |
| - | - | 12 | - | - | - | - | - | - |

**Supplemental Table 10: GSIS of encapsulated Canine Islets.**

Approximately 10 microspheres were in low glucose for 2 hours. The 3 different microsphere groups were rinsed and incubated in 2.8, 16.7 or 28 mM containing 30 mM K^+^ as a secretagogue for 90 minutes. Aliquots were removed and analyzed with a canine insulin ELISA.

| Glucose Concentrations (mM) | | | |
| --- | --- | --- | --- |
|  | 2.8 | 16.7 | 28t |
| Rep 1 | 1638 | 3362 | 6722 |
| Rep 2 | 835 | 2225 | 3515 |
| Rep 3 | 2554 | 1802 | 2446 |

**Supplemental Table 11: Body Weights.**

|  | Body Weight (g) at Weeks from Transplant | | | | | | | | | | | |
| --- | --- | --- | --- | --- | --- | --- | --- | --- | --- | --- | --- | --- |
| Dog ID | -1 week | 0 | 1 | 2 | 3 | 4 | 5 | 6 | 7 | 8 | 9 | 10 |
| 1 | 13.6 | 13.6 | 12.5 | 13.0 | 13.0 | 13.0 | - | - | - | - | - | - |
| 2 | 12.7 | 12.7 | 12.0 | 12.0 | 12.0 | 12.0 | - | - | - | - | - | - |
| 3 | 11.0 | 10.0 | 12.0 | 11.0 | - | - | - | - | - | - | - | - |
| 4 | 10.0 | 10.0 | 10.0 | 10.0 | - | - | - | - | - | - | - | - |
| 5 | 9.4 | 8.3 | 9.0 | 8.9 | 8.9 | 9.1 | 9.3 | 8.9 | 9.3 | 8.4 | 9.3 | 10.0 |
| 6 | 9.5 | 10.0 | 9.9 | 10.3 | 10.2 | 10.8 | 10.7 | 11.0 | 11.1 | - | - | - |
| 7 | 7.4 | 8.0 | 8.2 | 8.3 | 8.5 | 8.7 | 9.0 | 9.4 | 10.2 | - | - | - |

**Supplemental Table 12: Fasting Blood Glucose.**

Weekly fasting blood glucose was taken in the morning following an overnight fast. “Pre” indicates a measurement taken prior to the transplant.

| Weeks from Transplant | | | | | | | | | |
| --- | --- | --- | --- | --- | --- | --- | --- | --- | --- |
| Dog ID | Pre | 0 | 1 | 2 | 3 | 4 | 5 | 6 | 7 |
| 1 | 50 | 71 | 67 | 87 | - | - | - | - | - |
| 2 | 82 | 77 | 74 | 97 | - | - | - | - | - |
| 3 | 56 | - | 63 | 71 | 76 | 74 | - | - | - |
| 4 | 59 | - | 72 | 77 | 79 | 97 | - | - | - |
| 5 | 184 | 179 | 135 | 118 | 127 | 135 | 248 | 373 | 171 |
| 6 | 302 | 147 | 229 | 174 | 315 | 244 | 200 | 209 | 287 |
| 7 | 208 | 197 | 140 | 247 | 130 | 102 | 122 | 139 | 114 |

**Supplemental Table 13: Non-Fasting Blood Glucose.**

Veterinary staff collected blood twice daily immediately after the transplants and daily thereafter for blood glucose monitoring. The daily values were averaged and reported 2-3 times/week.

| Fasting Blood Glucose (mg/dL) by Dog ID | | | |
| --- | --- | --- | --- |
| Week | 5 | 6 | 7 |
| -1.0 | 208 ± 14 | 343 ± 8 | 289 ± 40 |
| -0.5 | 254 ± 41 | 387 ± 14 | 302 ± 95 |
| 0 | 292 ± 29 | 390 ± 57 | 221 ± 16 |
| 0.3 | 244 ± 70 | 172 ± 29 | 168 ± 21 |
| 0.6 | 134 ± 20 | 380 ± 68 | 155 ± 31 |
| 1.0 | 135 ± 10 | 292 ± 6 | 175 ± 19 |
| 1.2 | 148 ± 22 | 348 ± 51 | 154 ± 40 |
| 1.5 | 176 ± 77 | 275 ± 8 | 183 ± 38 |
| 1.7 | 150 ± 43 | 267 ± 8 | 153 ± 25 |
| 2.0 | 142 ± 14 | 293 ± 25 | 188 ± 20 |
| 2.3 | 118 ± 11 | 318 ± 60 | 262 ± 43 |
| 2.6 | 132 ± 3 | 300 ± 40 | 194 ± 50 |
| 3.0 | 168 ± 22 | 387 ± 26 | 191 ± 66 |
| 3.3 | 167 ± 14 | 298 ± 42 | 145 ± 8 |
| 3.6 | 208 ± 14 | 301 ± 24 | 119 ± 3 |
| 4.0 | 372 ± 18 | 288 ± 30 | 118 ± 7 |
| 4.5 | 238 ± 27 | 312 ± 62 | 103 ± 2 |
| 5.0 | 387 ± 14 | 424 ± 5 | 117 ± 11 |
| 5.5 | 342 ± 62 | 259 ± 12 | 157 ± 28 |
| 6.0 | 335 ± 2 | 298 ± 48 | 97 ± 15 |
| 6.5 | 398 ± 15 | 219 ± 7 | 122 ± 15 |
| 7.0 | 402 ± 3 | 202 ± 27 | 116 ± 18 |
| 7.3 | 371 ± 13 | 243 ± 2 | - |
| 7.6 | 443 ± 35 | 330 ± 55 | - |

**Table 14: Blood Chemistry Values**

Blood chemistry tests were conducted by a 3^rd^ party contract research organization. In several cases the diabetic dogs had abnormal blood chemistry values that normalized after the transplants. It’s also important to note that many veterinarians do not closely ascribe to the reference values for dogs as they do not have the same repeatability as humans.

NT = Not Tested , Bold values are out-of-range

| **Test** | **Normal Range** | **Dog ID** | **Pre** | **1 wk post** | **Termination** |
| --- | --- | --- | --- | --- | --- |
| ALP  (U/L) | 5-160 | 1  2  3  4  5  6  7 | 10  18  51  26  ***162***  ***175***  ***224*** | 65  53  53  32  94  108  123 | ***276***  51  44  32  101  83  105 |
| AST  (U/L) | 16-55 | 1  2  3  4  5  6  7 | 49  47  30  46  46  40  40 | ***68***  20  23  21  55  26  46 | ***184***  22  26  27  51  29  43 |
| ALT  (U/L) | 18-121 | 1  2  3  4  5  6  7 | ***8***  22  21  25  ***184***  ***148***  ***439*** | ***268***  18  29  24  87  45  98 | ***731***  15  24  26  79  30  63 |
| Creatine kinase  (U/L) | 10-200 | 1  2  3  4  5  6  7 | ***534***  ***586***  ***285***  ***746***  NT  NT  NT | ***274***  ***238***  194  ***346***  NT  NT  NT | 150  135  137  141  NT  NT  NT |
| Total bilirubin  (mg/dL) | 0.0-0.3 | 1  2  3  4  5  6  7 | ***0.4***  ***0.6***  0.2  ***0.7***  0.2  0.2  0.1 | ***0.5***  0.3  0.2  ***0.5***  0.2  0.2  0.2 | ***0.5***  0.2  0.3  0.3  0.2  0.2  0.2 |
| Bilirubin conjugated  (mg/dL) | 0.0-0.1 | 1  2  3  4  5  6  7 | 0.0  0.1  0.1  0.0  0.0  0.0  0.0 | ***0.2***  0.1  0.1  0.1  0.0  0.0  0.0 | 0.1  0.1  0.1  0.1  0  0  0 |
| Bilirubin unconjugated  (mg/dL) | 0.0-0.2 | 1  2  3  4  5  6  7 | ***0.4***  ***0.5***  0.1  ***0.6***  NT  NT  NT | 0.2  0.2  0.1  0.2  NT  NT  NT | ***0.6***  0.1  0.2  0.1  NT  NT  NT |
| Albumin  (g/dL) | 2.7-3.9 | 1  2  3  4  5  6  7 | 3.5  3.0  3.0  3.7  3.1  2.9  3.2 | 3.1  2.8  3.3  3.5  3.2  2.8  2.9 | 2.9  3.0  3.4  3.6  3.8  3.2  2.8 |
| Globulin  (g/dL) | 2.4-4.0 | 1  2  3  4  5  6  7 | 2.8  ***2.2***  2.8  3.0  2.7  2.7  2.6 | 3.1  3.2  2.9  2.7  2.7  2.9  3.0 | 3.2  3.0  3.2  2.6  3.8  3.2  2.8 |
| Cholesterol  (mg/dL) | 131-345 | 1  2  3  4  5  6  7 | 173  176  186  228  NT  NT NT | 212  248  219  241  NT  NT  NT | 258  221  225  243  NT  NT NT |
| Total Protein  (g/dL) | 5.5-7.5 | 1  2  3  4  5  6  7 | 6.3  ***5.2***  5.8  6.7  5.9  5.6  5.7 | 6.2  6.0  6.2  6.2  5.7  6.0  6.0 | 6.1  6.0  6.6  6.2  6.5  6.1  5.7 |
| BUN  (g/dL) | NA | 1  2  3  4  5  6  7 | 24  9  10  10  10  8  14 | 11  16  11  11  14  14  11 | 17  14  14  16  20  18  11 |
| Creatinine  (mg/dL) | 0.5-1.5 | 1  2  3  4  5  6  7 | 1.0  0.5  0.5  ***0.3***  0.8  ***0.4***  0.6 | 0.5  ***0.4***  0.5  ***0.4***  0.6  0.7  0.7 | 0.7  ***0.4***  0.5  ***0.4***  0.6  0.8  0.6 |
| Calcium  (mg/dL) | 8.8-11.2 | 1  2  3  4  5  6  7 | ***7.5***  ***6.4***  ***7.3***  ***6.2***  11.2  10.7  10.5 | 9.0  8.9  9.6  9.3  10.0  10.2  10.0 | 9.7  10.0  10.3  10.1  10.1  10.3  9.8 |
| Phosphorus  (mg/dL) | 2.5-6.1 | 1  2  3  4  5  6  7 | 5.0  3.9  3.7  4.7  5.9  5.5  6.5 | 4.2  4.4  3.7  3.6  5.9  5.6  ***6.7*** | 4.2  3.6  3.3  4.5  5.0  5.0  5.4 |
| Bicarbonate (mmol/L) | 13-27 | 1  2  3  4  5  6  7 | 20  21  25  21  NT  NT  NT | 19  21  22  20  NT  NT  NT | 19  24  26  25  NT  NT  NT |
| Chloride  (mmol/L) | 108-119 | 1  2  3  4  5  6  7 | 110  110  ***107***  ***107***  109  108  110 | 112  112  108  ***107***  ***106***  ***107***  109 | 115  112  ***107***  ***104***  ***105***  ***104***  110 |
| Potassium  (mmol/L) | 4.0-4.5 | 1  2  3  4  5  6  7 | ***5.5***  ***5.0***  ***5.0***  ***5.3***  ***4.7***  ***3.9***  4.5 | ***5.1***  ***4.6***  ***4.7***  ***4.7***  4.0  4.1  4.3 | ***5.1***  4.4  4.5  4.4  ***5.1***  4.4  4.3 |
| Sodium (mmol/L) | 142-152 | 1  2  3  4  5  6  7 | 146  145  145  145  146  147  147 | 144  145  143  143  144  143  143 | 144  146  146  143  141  141  147 |

**Table 15: Hematology Values.**

Blood samples were sent to a 3^rd^ party independent contract research organization for hematological testing. Values outside the normal range or in bold.

| **Test** | **Normal Range** | **Dog ID** | **Pre** | **1 week post** | **Termination** |
| --- | --- | --- | --- | --- | --- |
| WBC | 4.9-17.6 | 1  2  3  4  5  6  7 | 8.1  5.6  10.1  5.4  9.1  8.5  7.5 | 8.3  8.4  9.5  7.47  11.8  12.3  7.7 | 9.6  7.5  9.6  7.3  11.3  11.1  7.7 |
| RBC | 5.39-8.70 | 1  2  3  4  5  6  7 | 7.52  7.38  7.21  8.15  6.60  6.46  7.40 | 7.26  6.11  6.61  7.14  6.80  6.61  8.02 | 7.66  7.01  7.64  7.55  5.77  7.09  7.47 |
| HGB | 13.4-20.7 | 1  2  3  4  5  6  7 | 17.2  17.5  16.7  18.9  14.6  14.7  16.1 | 16.8  14.3  15.4  17.3  15.1  14.8  18.5 | 17.6  16.7  17.8  18.3  15.6  16.8  17.4 |
| HCT | 38.3-56.5 | 1  2  3  4  5  6  7 | 50.5  50.8  47.7  55.7  46.7  45.4  51.1 | 48.5  41.8  44.5  48.6  48.2  46.5  55.8 | 50.0  49.2  52.6  53.9  40.6  49.8  51.8 |
| MCV | 59-76 | 1  2  3  4  5  6  7 | 67  69  66  68  71  71  69 | 67  68  67  68  71  70  70 | 65  70  69  71  70  70  69 |
| MCH | 21.9-26.1 | 1  2  3  4  5  6  7 | 23.9  23.7  23.2  23.2  22.1  22.7  ***21.8*** | 23.1  23.4  23.3  24.2  22.2  22.5  ***21.8*** | 23.0  23.8  23.3  24.2  27.0  23.6  23.3 |
| MCHC | 32.6-39.2 | 1  2  3  4  5  6  7 | 34.1  34.4  35.0  33.9  ***31.2***  ***32.3***  ***31.5*** | 34.6  34.2  34.6  35.6  ***31.3***  ***31.9***  ***31.4*** | 35.2  33.9  33.8  34.0  38.4  33.6  33.6 |
| Neutrophils | 2940-12670 | 1  2  3  4  5  6  7 | 5800  3489  7262  2078  5900  4700  3750 | 6059  5544  6574  5051  8890  7830  3970 | 7046  5430  6720  5103  7750  6830  3610 |
| Monocytes | 130-1150 | 1  2  3  4  5  6  7 | 243  168  242  108  750  620  590 | 274  218  418  169  ***1200***  870  630 | 208  156  243  189  940  670  1150 |
| Lymphocytes | 1060-4950 | 1  2  3  4  5  6  7 | 1920  1775  2030  2160  2280  2890  2790 | 1760  2066  1872  2325  2100  3030  2600 | 1997  1650  1803  2570  2220  3100  2490 |
| Eosinophils | 70-1490 | 1  2  3  4  5  6  7 | 130  157  556  ***54***  130  230  240 | 191  563  618  123  410  530  290 | 173  210  480  190  290  410  330 |
| Basophils | 0-100 | 1  2  3  4  5  6  7 | 8  11  10  0  30  40  80 | 17  8  19  31  50  80  80 | 29  8  10  7  40  90  80 |
| Coagulation – Fibrinogen | 90-255 | 1  2  3  4  5  6  7 | 208  189  151  95  ***335***  196  195 | 180  ***393***  ***352***  198  ***377***  ***382***  ***276*** | 220  241  214  159  ***324***  186  ***261*** |
